# Disparities in healthy food zoning, farmers’ market availability, and fruit and vegetable consumption among North Carolina residents

**DOI:** 10.1186/s13690-015-0085-9

**Published:** 2015-08-25

**Authors:** Stephanie Bell Jilcott Pitts, Mariel Leah Mayo Acheson, Rachel K Ward, Qiang Wu, Jared T McGuirt, Sally L Bullock, Mandee F Lancaster, Justin Raines, Alice S Ammerman

**Affiliations:** Department of Public Health, Brody School of Medicine, East Carolina University, 600 Moye Blvd, MS 660, Lakeside Annex 7, Greenville, NC 27834 USA; Department of Community Health, East Tennessee State University, Johnson City, TN USA; Department of Biostatistics, East Carolina University, 2435D Health Sciences Building, Greenville, NC 27834 USA; Department of Nutrition, University of North Carolina at Chapel Hill, Chapel Hill, NC 27599 USA; Center for Survey Research, Office of Innovation and Economic Development, East Carolina University, Greenville, NC USA; Department of Nutrition, Gillings School of Global Public Health, Center for Health Promotion and Disease Prevention, University of North Carolina at Chapel Hill, CB# 7426, Chapel Hill, NC 27599-7426 USA

**Keywords:** Disparities, Farmers’ market, Zoning ordinance

## Abstract

**Background:**

Context and purpose of the study. To examine (1) associations between county-level zoning to support farmers’ market placement and county-level farmers’ market availability, rural/urban designation, percent African American residents, and percent of residents living below poverty and (2) individual-level associations between zoning to support farmers’ markets; fruit and vegetable consumption and body mass index (BMI) among a random sample of residents of six North Carolina (NC) counties.

**Methods:**

Zoning ordinances were scored to indicate supportiveness for healthy food outlets. Number of farmers’ markets (per capita) was obtained from the NC-Community Transformation Grant Project Fruit and Vegetable Outlet Inventory (2013). County-level census data on rural/urban status, percent African American, and percent poverty were obtained. For data on farmers’ market shopping, fruit and vegetable consumption, and BMI, trained interviewers conducted a random digit dial telephone survey of residents of six NC counties (3 urban and 3 rural). Pearson correlation coefficients and multilevel linear regression models were used to examine county-level and individual-level associations between zoning supportiveness, farmers’ market availability, and fruit and vegetable consumption and BMI.

**Results:**

At the county-level, healthier food zoning was greater in more urban areas and areas with less poverty. At the individual-level, self-reported fruit and vegetable consumption was associated with healthier food zoning.

**Conclusions:**

Disparities in zoning to promote healthy eating should be further examined, and future studies should assess whether amending zoning ordinances will lead to greater availability of healthy foods and changes in dietary behavior and health outcomes.

## Background

Obesity and related chronic diseases are major public health concerns [1, 2], with higher rates in rural versus urban areas [3–5]. Encouraging consumption of fruits and vegetables, as a substitute for higher-calorie, processed foods, is thought to lower obesity and related chronic disease risk and disparities [6–9], yet United States residents [10], particularly rural residents [11], do not consume recommended amounts. Improving access to healthy food outlets, such as placing farmers’ markets in communities, is one strategy to increase consumption of healthy foods [12]. This strategy is supported by evidence that individuals who live closer to farmers’ markets are less likely to be obese [13, 14] and those who shop at farmers’ markets report consuming more fruits and vegetables than those who do not [15–17].

Some maintain that the connection between planning and public health had its roots in the landmark 1926 Supreme Court case *Village of Euclid v. Ambler Realty,* which helped establish local zoning of land uses in municipalities across the United States [18]. Local planning and zoning policies can promote or hinder the establishment and sustainability of healthy food outlets [19]. For example, in Mecklenberg County, North Carolina (NC), zoning ordinances posed major obstacles to establishing mobile farmers’ markets [20]. Not much is known about whether zoning to support healthy food outlets varies by rural/urban designation or poverty levels. If such disparity exists, it could negatively impact access to healthy food in rural and low-income areas [19], potentially contributing to existing health disparities.

To increase access to fresh produce in underserved areas between the years of 2012-2014, the NC Community Transformation Grant Project (CTG-Project) promoted state-wide efforts to enhance farmers’ markets through marketing, promotion, improving transportation, and amending zoning ordinances to be more supportive of farmers’ markets [21]. For example, efforts of the NC CTG-Project facilitated ordinance amendments in some communities to allow farmers’ markets in all zoned districts. The assumption underlying such efforts is that zoning modifications will lead to greater availability of farmers’ markets, as well as more fruit and vegetable purchasing and consumption. To examine the validity of this assumption and the potential effectiveness of zoning modifications, it is important to learn if zoning to support farmers’ markets is associated with greater availability of farmers’ markets (more farmers’ markets per capita in the zoned jurisdiction), more farmers’ market shopping, and greater fruit and vegetable consumption. In Northeastern NC, more supportive zoning was associated with more farmers’ markets [22]. However, to our knowledge, no studies have examined associations between zoning to support healthy food retail, availability of farmers’ markets, and residents’ eating behaviors and weight status.

Therefore, we examined (1) county-level associations between zoning to support farmers’ markets, availability of farmers’ markets, rural/urban designation, percent African American residents, and percent of residents living below poverty among 33 NC counties and (2) individual-level associations between zoning to support farmers’ markets, farmers’ market shopping, self-reported fruit and vegetable consumption and body mass index (BMI) among a random sample of residents (*n* = 615) in three urban and three rural NC counties. Our hypotheses were that (1) counties with more supportive zoning ordinances would be more urban, have a lower percent of African American residents, and a lower proportion of residents living below poverty (due to potentially having more zoning-related resources at the county level); and (2) more supportive zoning would be associated with more farmers’ market shopping, greater produce consumption, and lower BMI.

## Methods

### Study overview

Between 2012-2014, the Community Transformation Grant (CTG) Program was funded by the Affordable Care Act’s Prevention and Public Health Fund, through which the Centers for Disease Control and Prevention supported US communities to implement chronic disease prevention interventions. The NC CTG-Project included a focus on farmers’ market enhancements and promotion in 98 of NC’s 100 Counties (two urban counties were not included). The enhancements and promotions included updating land use plans and zoning ordinances to increase support for farmers’ markets. It was anticipated that such updates would lead to greater access to farmers’ markets, as well as more fruit and vegetable purchasing and consumption. The current study sought to examine this expected outcome in six counties in three NC CTG-Project regions. This study was reviewed and approved by the East Carolina University Institutional Review Board.

### Study setting and participants

To represent geographic diversity within NC, we selected counties in each of three distinct geographic regions of North Carolina: the Western mountains, Central piedmont, and Eastern coastal plain. Within each region, we selected one rural and one urban county in which to conduct a random-digit-dial (RDD) survey about farmers’ market shopping, similar to a previous RDD survey [17]. For the county-level analyses, the sample included 33 counties in the three regions for which we obtained and coded zoning ordinances. For the individual-level analyses, the sample included 615 RDD respondents who were residents of the six counties of interest. We aimed for 100 respondents in each of the six counties.

### Healthy outlet zoning scores

First, zoning ordinances were obtained and scored for the municipalities in each region. As in prior work [22], we used sections of the Bridging the Gap Community Obesity Measures Project (BTG-COMP) food code/policy audit form [23] to code and score available county and municipality zoning ordinances. We conducted internet searches to find the most recently available zoning ordinances between June 2013 and October 2013. Graduate research assistants contacted local planners to obtain paper copies of ordinances that were not available online, but were unsuccessful.

The Western Mountain Region includes 10 counties, 32 municipalities, and 37 county and/or municipality zoning ordinances that were coded and scored. The Central Piedmont Region includes 10 counties, 37 municipalities, and 46 zoning ordinances that were coded and scored. The Coastal Plain Region includes 15 counties (only 13 were included, as two did not have zoning ordinances), 17 municipalities, and 28 zoning ordinances that were coded and scored as detailed elsewhere [22].

The county and municipality zoning ordinances were coded by two individual coders using the Bridging the Gap Community Obesity Measures Project (BTG-COMP) food code/policy audit form. First, the coders determined if any of the seven possible zoning districts (i.e., agriculture, code reform, commercial, mixed use, public/civic/government/school, recreation/open space, and residential) from the BTG-COMP food code/policy audit form were included in the zoning ordinances. A zoning district is a specified area of land in a county or municipality where certain specific uses are allowed. In many cases, the county zoning ordinance applies to any *non-zoned areas* of the county, and municipality (city or town within a county) ordinances only apply only to a *specific municipality*.

For each county or municipality zoning ordinance, the coders reviewed each of the six food outlet subsections included in the audit form (i.e., farmers’ markets, green/fresh fruit and vegetable carts, mobile food vendors/carts, urban agriculture, produce/fresh fruit and vegetable stands, and produce/fruit market/stores), and assigned one point if the food outlet was addressed in any of the seven possible zoned districts. If the type of food outlet (e.g., farmers’ markets, green/fresh fruit and vegetable carts, etcetera) was addressed, then points were assigned based on the range of allowed uses in that district. Specifically, four points were assigned if the use was permitted (allowed without conditions), three points if the permitted use was conditional, two points if the use was accessory (or secondary to the primary land use), one point if use was prohibited, and zero points if the use type was not specified. Finally, after independently coding the ordinances, the two coders met to compare assigned points and come to an agreement for the final coding decisions.

For individual-level analyses, each county and municipality zoning ordinance was then assigned an un-weighted Healthy Outlet Zoning Score [22] that addressed the aspects of the zoning ordinance related to healthy food outlets. These included the six subsections: farmers’ markets, green/fresh fruit and vegetable carts, mobile food vendors/carts, urban agriculture, produce/fresh fruit and vegetable stands, and produce/fruit market/stores. The *un-weighted Healthy Outlet Zoning Score* was calculated as the total number of points assigned to its ordinance, divided by the highest or maximum number of points that could be assigned to the subsections based on the number of zoning districts coded. For example, in County X, there were four districts present: agricultural, commercial, mixed-use, and residential. Only three subsections were addressed in the ordinance: farmers’ markets, urban agriculture, and produce/fruit market/stores. Within each of the four districts present in County X, farmers’ markets were permitted (allowed without conditions) in two districts (4 + 4 = 8), and conditional (allowed pending some conditions) in two other districts (3 + 3 = 6). Thus, the sub-total for farmers’ markets was 14 + 4 (because farmers’ markets were addressed in four districts) = 18. Urban agriculture was permitted in all four districts without conditions (4 × 4 = 16), so the sub-total for urban agriculture was 16 + 4 = 20. Produce/fruit markets/stores allowances were identical to farmers’ markets, so the sub-total for produce/fruit markets/ stores was 18. Thus, the points assigned to County X were 18 + 20 + 18 = 56. In County X, the maximum number of points assigned would be the number of districts (four in this case), multiplied by six (the number of sub-sections), multiplied by four (to represent the ‘permitted use’ or the ideal situation or the “healthiest zoning possible”), or 4 × 6 × 4 = 96. So the final un-weighted score would be 56 (total points)/96 (maximum possible points) = 0.58.

The un-weighted Healthy Outlet Zoning Score (either at the county or municipality level) was used in the individual-level analyses using the six-county RDD survey data. Each RDD respondent was assigned the Healthy Outlet Zoning Score corresponding to the municipality (or county) of the residential location. We assigned the municipality scores to each individual, because we needed a proxy for individual-level exposure to municipality policies. The only case in which the un-weighted Healthy Outlet Zoning Score at the county-level was assigned to the individual was if the respondents’ residential location was in a municipality that did not have a zoning ordinance. In those cases, no municipality-level Healthy Outlet Zoning Score could be computed.

For the county-level analyses, the weighted county-level Healthy Outlet Zoning Score was the weighted sum of the county and its respective municipality(s) un-weighted scores, derived by multiplying the un-weighted score by the proportion of the total county population residing in the county or the municipality, and then summing these scores. For example, if County *X* has two municipalities (Municipality *Y* and Municipality *Z*), if 20 % and 30 % of county residents live in those two municipalities, respectively, and the un-weighted score for Municipality *Y* is 0.125, Municipality *Z* is 0.250, and the un-weighted county score is 0.208, then the weighted county level Healthy Outlet Zoning Score 1 was calculated as (0.20 × 0.125) + (0.30 × 0.250) + (0.50 × 0.208) = 0.204. Healthy Outlet Zoning Scores ranged from 0-1, with a higher score representing healthier food zoning.

### County-level availability of Farmers’ markets

We defined farmers’ markets as “*a venue with a predictable location and hours of operation that sells produce, but that is not a retail store*.” We defined “Availability of Farmers’ Markets” as the number of markets in 33 counties of interest divided by the county population, to obtain a per capita farmers’ market measure. To determine the number of farmers’ markets in each county, we used data from the NC-CTG Project Fruit and Vegetable Outlet Inventory, 2013, which was designed to organize data regarding farmers’ market locations, amenities, and enhancements, and collected by NC CTG-Project staff. These data were compiled by the evaluation team and the number of farmers’ markets in each of the 33 counties was counted, divided by county population, and used in county-level analyses.

### County-level rural/urban designation, percent African american residents, and percent residents living below poverty

We categorized counties as rural in two ways. First, the 2013 Urban Influence Codes were used. These codes classify metro (or urban) counties into two categories based upon the size of their metro area and non-metro (or more rural) counties into 10 categories based upon proximity to metro areas and the size of the largest city or town in the county [24]. We also used the percentage of the total county population residing in a rural area. [25] Using Census data, we determined the percentage of African American residents in each county [26]. Finally, as a measure of county-level economic status, we used percentage of county residents living in poverty in 2012, as defined by the Census Bureau [27].

### Random digit dial survey administration

Between June and October 2013, trained interviewers conducted a six-county RDD telephone survey. Land lines (*n* = 8,697) and cellular telephone lines (*n* = 7,006) were included in the purchased sample provided by Survey Sampling International (http://www.surveysampling.com/), and numbers were called during a variety of days and times. Eligibility criteria included being over 18 years of age, a resident of one of the six counties, and one of the primary food shoppers in the household. The adult who answered the phone and met the eligibility criteria was interviewed. Up to 10 attempts were made to each number in the sample. In addition, up to 5 scheduled callbacks were made to those reached at an inconvenient time or did not answer the phone, and one conversion was attempted for each soft refusal. Of 12,025 phone numbers contacted, there were 615 surveys completed, 2794 refusals, and 8616 not eligible due to language barriers, numbers not in service, not residents of the county of interest, business numbers, no answer or no adult being home. Between 100 and 108 residents completed the survey from each county. The final response rate was 18 %. The completed interview lasted between 25 and 30 min. A $10 gift card incentive was mailed to the participant’s home address upon survey completion.

### Farmers’ market shopping frequency

RDD survey participants were asked “How often in the past 12 months did you buy fruits or vegetables locally grown such as from a farmers’ market, CSA, roadside stand, or pick-your-own produce farm?” Because of the distribution of responses, responses were dichotomized into never versus ever shopping at farmers’ markets, which was used as both an independent and dependent variable in separate analyses.

### Fruit and vegetable consumption and body mass index (BMI)

Self-reported fruit and vegetable consumption and BMI were dependent variables. Fruit and vegetable consumption was measured in two ways. The first used responses to the Block Fruit, Vegetable, and Fiber Screener items, and was scored using the standard equations and converted to fruit and vegetable servings consumed per day (hereafter referred to as “Block Fruit and Vegetable Consumption”) [28, 29] The second approach used responses to questions taken from the Behavioral Risk Factor Surveillance System, which included: “During the past month, not counting juice, how many times per day, week, or month did you eat… fruit?; dark green vegetables?; orange colored vegetables?; and other vegetables?” Responses to these items were converted into times per day and summed to create a separate fruit and vegetable consumption variable (hereafter referred to as “BRFSS Fruit and Vegetable Consumption”). The correlation between the Block and BRFSS Fruit and Vegetable Consumption variables was 0.34 (*p* < 0.001). BMI was calculated from self-reported height and weight and corrected for systematic error using age, race and gender [30].

### Data analysis

For our county-level analyses, we examined Pearson’s correlation coefficients between the weighted county Healthy Outlet Zoning Scores, number of farmers’ markets, percent rural residents, percent African American residents, and percent of residents living below the federal poverty level.

For the individual-level analyses, the Healthy Outlet Zoning Score was considered the exposure, and we examined the cross-sectional association between exposure to the Zoning Score and farmers’ market shopping, fruit and vegetable consumption and BMI. We first examined the association between Healthy Outlet Zoning Scores (independent variable) and farmers’ market shopping (dependent variable) using adjusted logistic regression models.

Next, we used multi-level models to examine the association between fruit and vegetable consumption and BMI (dependent variables), and Healthy Outlet Zoning Scores and farmers’ market shopping (independent variables). The Healthy Outlet Zoning Scores was modeled as a random effect which might interact with other fixed effects such as farmers’ market shopping. The purpose of these models was to test the heterogeneity in the fixed effects due to differing zoning scores. However, for the models with fruit and vegetable consumption as the dependent variable, the multi-level models identified no variance for the random zoning score effects; thus, these models were essentially reduced to fixed effects models with an interaction term.

Three multilevel models were used to examine associations between the dependent variables of self-reported fruit and vegetable consumption and BMI and the independent variables of the Healthy Outlet Zoning Score and farmers’ market shopping (never versus ever). All models were adjusted by factors including age (years), sex (male vs. female), race (white vs. others), and graduated from college (yes vs. no), which may all be associated with farmers’ market shopping, fruit and vegetable consumption and weight status. Because county-level rural or urban status may influence the relationship between zoning scores, farmers’ market shopping, and self-reported fruit and vegetable consumption, we included an interaction term between zoning scores and the Office of Management and Budget dichotomous measure of metro versus non-metro counties. Subject to missing values, each model used 517-564 observations. All statistical analyses were conducted in SAS version 9.3 (SAS Institutes, Cary, North Carolina).

## Results and discussion

County (*n* = 33) zoning scores, percent poverty, percentage of rural residents, and percentage of African American residents are reported in Table 1. There was a mean of 6 farmers’ markets per county. The county-level mean percentage of rural residents was 65 %, 21 % below poverty, 25 % African American residents, and a mean of 31 % of obese adults.

**Table 1:** County Characteristics of 33 regionally diverse North Carolina Counties

Characteristic	Mean	Standard Deviation	Minimum	Maximum
Number of farmers’ markets per 10,000 capita, 2013	1.46	1.06	0	5.20
Number of farmers’ markets with Supplemental Nutrition Assistance Program Electronic Benefit Transfer per 10,000 capita, 2013	0.21	0.28	0	0.82
Urban Influence Code, 2013	4.42	2.85	1.00	11.00
Percentage of population in rural areas	65.06	24.40	7.35	100.00
Percentage of population living below poverty level	20.83	5.81	9.70	31.80
Percentage of African American residents	24.78	19.92	0.90	61.90
County population	63,348	78,926	9,980	35,0670
Percentage of adult residents with diabetes	12.19	1.71	9.20	15.20
Percentage of adult residents with obesity	30.63	3.35	22.50	37.00
Percentage of children with obesity	15.08	2.68	8.00	21.10
County-level Healthy Outlet Zoning Score	0.34	0.22	0.02	0.83

The 2013 Urban Influence Codes were used to classify metro (or urban) counties into two categories based upon the size of their metro area and non-metro (or more rural) counties into 10 categories based upon proximity to metro areas and the size of the largest city or town in the county [24]. The County-level Healthy Outlet Zoning score was created using a weighted sum of municipality and county-level zoning ordinances coded using the Bridging the Gap Community Obesity Measures Project (BTG-COMP) food code/policy audit form [22]

There were no associations between county-level zoning to support farmers’ markets (Healthy Outlet Zoning Score) and number of farmers’ markets in the 33 NC counties (*r* = 0.035, *p* = 0.85). There was a moderate, inverse association between Healthy Outlet Zoning Scores and urban influence codes that approached statistical significance—indicating healthier food zoning in more urban areas (*r* = -0.333, *p* = 0.058). There was an inverse association between Healthy Outlet Zoning Scores and percent poverty—indicating healthier food zoning in areas with less poverty (*r* = -0.381, *p* = 0.029). There were no other statistically significant correlations between healthy food zoning and rural/urban or percent African American residents (Table 2).

**Table 2:** Pearson’s correlation coefficients between county-level Healthy Outlet Zoning Score* and county-level characteristics (*N* = 33 counties)

Characteristics	Correlation between each characteristic and county-level zoning to support farmers’ markets (Healthy Outlet Zoning Score)	*p*-value
Farmers’ markets per 10,000 capita, 2013	0.19	0.30
Farmers’ markets with SNAP/EBT per 10,000 capita, 2013	0.09	0.64
Urban influence codes, 2013	−0.33	0.06
Percent rural population	0.04	0.83
Percent of residents living under the federal poverty level	−0.38	0.03
Percent of African American residents	−0.02	0.90

*Healthy Outlet Zoning Scores ranged from 0-1, with a higher score representing healthier food zoning

Table 3 shows individual characteristics of random digit dial respondents (18 % response rate). Over sixty percent were white, 53 % were college educated, and 76 % were female. The sample had a mean age of 55 years, mean corrected BMI of 30 kg/m^2^, consumed a mean of 3.7 fruits and vegetables per day according to the Block Fruit and Vegetable Screener and 2.9 fruits and vegetables per day according to the BRFSS Fruit and Vegetable Consumption.

**Table 3:** Random digit dial respondent characteristics in six North Carolina counties

Characteristic	N	Frequency	Percent
Race	608		
White		372	61.18
Other		236	38.82
Education	599		
College Graduate		320	53.42
Other		279	46.58
Sex	613		
Male		149	24.31
Female		464	75.69
Characteristic	N	Mean	Standard Deviation
Age	593	55.4	17.0
BMI	564	28.8	6.3
BMI Corrected	535	30.0	6.7
Block Fruit and Vegetable Consumption, servings per day	610	3.7	1.9
Behavioral Risk Factor Surveillance System (BRFSS) Fruit and Vegetable Consumption, servings per day	570	2.9	2.4

At the individual-level, there was no association between farmers’ market shopping and Healthy Outlet Zoning Score. Table 4 presents results from three multi-level models: There was a statistically significant association between Block Fruit and Vegetable Consumption (servings per day) and shopping at a farmers’ market (*b* = -0.80 (0.28), *p* < 0.0043), such that more fruit and vegetable consumption was associated with shopping at farmers’ markets. There were no associations between Block Fruit and Vegetable Consumption and Healthy Outlet Zoning Scores.

**Table 4:** Associations between farmers’ market shopping, fruit and vegetable consumption, healthy zoning, and BMI among random digit dial survey participants (NC residents)

Model Number	Dependent variable	Independent variable	Beta estimate (Standard error)	*p*-value
1	Block Fruit and Vegetable Consumption (servings per day)	Farmers’ market shopping (never versus ever)	−0.80 (0.28)	0.0043
		Healthy Outlet Zoning Scores	0.72 (1.68)	0.67
2	BRFSS Fruit and Vegetable Consumption (servings per day)	Farmers’ market shopping (never versus ever)	0.31 (0.39)	0.43
		Healthy Outlet Zoning Scores	5.42 (2.40)	0.02
3	Corrected BMI (kg/m^2^)	Farmers’ market shopping (never versus ever)	−1.03 (1.08)	0.34
		Healthy Outlet Zoning Scores	−12.26 (7.58)	0.11

Subject to missing values, each model included 517-564 observations. Dependent variables are fruit and vegetable consumption (self-reported from both the Block Fruit and Vegetable Screener and Behavioral Risk Factor Surveillance System (BRFSS) questions) and corrected body mass index (BMI). All models are multi-level, adjusted for age, race, sex, educational level, and county-level metro status

Conversely, BRFSS Fruit and Vegetable Consumption (servings per day) was positively associated with the Healthy Outlet Zoning Score (*b* = 5.42, *p* = 0.02). Farmers’ market shopping was not significantly associated with BRFSS Fruit and Vegetable Consumption when the Healthy Outlet Zoning Score was zero (*b* = 0.31, *p* = 0.43), but as the Healthy Outlet Zoning Score increased, the effect of farmers’ market shopping increased significantly at a rate of 2.72 (*p* = 0.01) per unit Healthy Outlet Zoning Score. In other words, when the Healthy Outlet Zoning Score was low, there was no association between self-reported fruit and vegetable consumption and farmers’ market shopping. However, as the zoning score increased, there was a strong, positive association between self-reported fruit and vegetable consumption and farmers’ market shopping.

In models which included the interaction term for county-level metro status, we found that the associations between Healthy Outlet Zoning Scores and (1) farmers’ market shopping (*p* for interaction = 0.02) and (2) self-reported Block Fruit and Vegetable Consumption (*p* for interaction = 0.0025) were lower in rural versus urban counties when the zoning score was zero, and as the zoning score increased, the gap between the rural and urban counties narrowed.

When the corrected BMI was used as the dependent variable, there was an inverse (though not statistically significant) association between Healthy Outlet Zoning Score and BMI (*b* = -12.26, *p* = 0.11). The association between BMI and farmers’ market shopping was not significant when the zoning score was zero (*b* = -1.03, *p* = 0.34) but as the zoning score increased, the association increased and was statistically significant (*b* = 6.64, *p* = 0.03). In other words, as the Healthy Outlet Zoning Score increased, there was an inverse association between shopping at farmers’ markets and BMI. No other associations reached statistical significance.

At the county-level, zoning to support farmers’ markets and farmers’ markets per capita (farmers’ market availability) were not associated, contrary to our hypothesis and previously published findings [22]. This may be due in part to the lag time needed between zoning ordinance adoption and translation into actual changes in the community food environment. This may also be due to variation in the ways individual municipalities act upon zoning ordinances. There may be supply and demand imbalances that prevent the establishment and maintenance of markets, even when the zoning is supportive.

There was an inverse, though not statistically significant, association between county-level Healthy Outlet Zoning Scores and urban influence codes, indicating healthier food zoning in more urban areas. We also found an inverse association between Healthy Outlet Zoning Scores and percent poverty, indicating healthier food zoning in areas with less poverty. Both of these associations may be due to the fact that urban and affluent areas might be more likely to have resources to support healthy zoning. Areas of higher poverty may have more residents with federal nutrition assistance benefits that could affect farmers’ market shopping and perhaps even zoning, although the direction of the effect would be difficult to estimate. Ultimately, if efforts are going to be made to modify zoning to support healthy food venues, it is necessary to know whether zoning changes translate into changes in the community food environment, and whether there are different effects of zoning based on contextual factors such as rurality and poverty. Our study was a first attempt at elucidating these associations, and further research is needed to determine zoning modifications that are most beneficial.

The Healthy Outlet Zoning Scores were positively associated with self-reported fruit and vegetable consumption and inversely associated with BMI. As the zoning scores increased, the positive association between farmers’ market shopping and self-reported fruit and vegetable consumption increased. Taken together, these results indicate that healthier food zoning is associated with healthier dietary behaviors. This suggests that CTG Project efforts to increase availability of farmers’ markets through amending land use plans and zoning ordinances may positively impact public health. However, we did not find that healthier zoning was associated with more farmers’ markets. This could be because healthier zoning is facilitating the development of other healthy food outlets (not just farmers’ markets) that were not counted as farmers’ markets in our availability measure (the NC Fruit and Vegetable Outlet Inventory). A simulation study based in an urban area (Pasadena, California) found that zoning to improve access to fruits and vegetables would increase produce consumption only marginally, with authors concluding that other environmental and policy levers (e.g., changing social norms related to healthy eating) would be more effective in improving dietary behaviors [31]. However, this study took place in an urban area, and more research is needed in both urban and rural areas to determine if healthier food zoning positively influences the community food environment and residents’ dietary behaviors.

The finding that fruit and vegetable consumption was associated with farmers’ market shopping could be due to farmers’ market shoppers perceiving they eat more fruits and vegetables because they make an effort to shop at a farmers’ market, and thus are systematically over-reporting consumption. In addition, farmers’ markets are often seasonal and the random digit dial survey was conducted in the summer and early fall; Thus, any true associations between fruit and vegetable consumption and farmers’ market shopping may be seasonal in nature. As we did not examine consumption of other foods, and as farmers’ markets may also sell less healthy, processed foods [32] individuals who shop at farmers’ markets may also be consuming more of these less healthy, processed foods in addition to fruits and vegetables.

This study has several limitations. One is the potential for systematic bias in the availability of zoning ordinances online (i.e., rural areas may be less likely to have their ordinances posted online). However, we attempted to contact county planning staff to obtain ordinances if we could not find them online. There is also lag time between ordinance adoption and establishment of new markets, which is a limitation of our cross-sectional analyses. Furthermore, our response rate (18 %) was low, especially compared to other random digit dial surveys [33, 34]. However, differences in how an eligible respondent is defined could lead to differences in calculated response rates even in the same study [35]. Our low response rate may indicate our sample is not representative of the residents of the counties surveyed. For example, our survey consisted of 61 % white respondents, while the county demographics indicate that 56 % of respondents should have been white if it were a truly representative sample. While we did not conduct a representative sample from each of the six counties, our goal was to obtain an equal sample size in both rural and urban areas, as rural areas are often not well represented in large community surveys such as the Behavioral Risk Factor Surveillance System Survey. Additionally, we used self-reported weight and height were used to determine BMI, and two self-reported measures of fruit and vegetable consumption, which were not highly correlated, and due to their self-reported nature, likely contained error. The BRFSS fruit and vegetable measure referenced consumption over the past month, which may lead to additional error. Finally, the NC CTG Project included promotional materials and other enhancements to increase shopping at farmers’ markets, which may confound our assessment of participants’ exposure to the policy and environmental structures related to farmers’ markets.

## Conclusions

Examining the potential for disparities in zoning ordinance enactment and translation to healthier food venues “on the ground” has not been widely studied, and the current study is one of the first attempts to do so. Such studies are needed to inform future efforts to reduce health disparities, including interventions targeting zoning modifications. Changes to zoning related to the food environment may help improve food access and reduce health disparities, particularly among disadvantaged populations.

## Abbreviations

NC: North Carolina
CTG-Project: NC Community Transformation Grant Project
RDD: Random-digit-dial
BTG-COMP: Bridging the Gap Community Obesity Measures Project
